# Single-Cell Transcriptome Analysis Reveals Different Immune Signatures in HPV- and HPV + Driven Human Head and Neck Squamous Cell Carcinoma

**DOI:** 10.1155/2022/2079389

**Published:** 2022-09-16

**Authors:** Simin Li, Yang Wang, Rui Sun, Debora Franceschi, Hongying Pan, Chenxuan Wei, Anthony Chukwunonso Ogbuehi, Bernd Lethaus, Vuk Savkovic, Sebastian Gaus, Rüdiger Zimmerer, Dirk Ziebolz, Gerhard Schmalz, Xiao Jiang

**Affiliations:** ^1^Stomatological Hospital, Southern Medical University, Guangzhou 510280, China; ^2^State Key Laboratory of Biocatalysis and Enzyme Engineering, School of Life Sciences, Hubei University, Wuhan, China; ^3^Department of Hernia and Abdominal Wall Surgery, Beijing Chaoyang Hospital, Capital Medical University, Beijing 100020, China; ^4^Department of Experimental and Clinical Medicine, University of Florence, Florence, Italy; ^5^School of Dentistry, University of Michigan, 1011 N University Ave, Ann Arbor, MI 48109, USA; ^6^Faculty of Physics, University of Münster, Wilhelm-Klemm-Straße 9, Münster 48149, Germany; ^7^Department of Cranio Maxillofacial Surgery, University Clinic Leipzig, Liebigstr 12, Leipzig 04103, Germany; ^8^Department of Cariology, Endodontology and Periodontology, University of Leipzig, 04103 Leipzig, Germany

## Abstract

**Background:**

Head and neck squamous cell carcinoma (HNSCC) is a significant health problem and related to poor long-term outcomes, indicating more research to be done to deeply understand the underlying pathways.

**Objective:**

This current study aimed in the assessment of the viral- (especially human papilloma virus [HPV]) and carcinogen-driven head and neck squamous cell carcinoma (HNSCC) microenvironment based on single-cell sequencing analysis.

**Methods:**

Data were downloaded from GEO database (GSE139324), including 131224 cells from 18 HP- HNSCC patients and 8 HPV+ HNSCC patients. Following data normalization, all highly variable genes in single cells were identified, and batch correction was applied. Differentially expressed genes were identified using Wilcoxon rank sum test. A gene enrichment analysis was performed in each cell cluster using KEGG analysis. Single-cell pseudotime trajectories were constructed with MONOCLE (version 2.6.4). Cell-cell interactions were analyzed with CellChat R package. Additionally, cell-cell communication patterns in key signal pathways were compared in different tissue groups. A hidden Markov model (HMM) was used to predict gene expression states (on or off) throughout pseudotime. Five-year overall survival outcomes were compared in both HPV+ and HPV- subsets.

**Results:**

20,978 high-quality individual cells passed quality control. RNA-seq data were used from 522 HNSCC primary tumor samples. 1,137 differentially expressed genes between HPV+ and HPV- HNSCC patients were investigated. 96 differentially expressed genes were associated with overall survival and highly enriched in B cell associated biological process. Cell composition differed between types of samples. MHC-I, MHC-II, and MIF signaling pathways were found to be most relevant. Within these pathways, some cells were either signal receiver or signal sender, depending on sample type, respectively. Six genes were obtained, AREG and TGFBI (upregulation), CD27, CXCR3, MS4A1, and CD19 (downregulation), whose expression and HPV types were highly associated with worse overall survival. AREG and TGFBI were pDC marker genes, CXCR3 and CD27 were significantly expressed in T cell-related cells, while MS4A1 and CD19 were mainly expressed in B naïve cells.

**Conclusions:**

This study revealed dynamic changes in cell percentage and heterogeneity of cell subtypes of HNSCC. AREG, TGFBI, CD27, CXCR3, MS4A1, and CD19 were associated with worse overall survival in HPV-related HNSCC. Especially B-cell related pathways were revealed as particularly relevant in HPV-related HNSCC. These findings are a basis for the development of biomarkers and therapeutic targets in respective patients.

## 1. Introduction

Head and neck squamous cell carcinoma (HNSCC) summarizes a group of malignant tumors, which originate from the mucosal epithelium in the oral cavity, pharynx, or larynx [1]. HNSCC, which is the most common malignancy of the head and neck, is related to different environmental factors, including tobacco and/or alcohol consumption [1]. Beside of these risk factors for HNSCC development, infections with the human papillomavirus (HPV) can induce oropharyngeal tumors; these malignancies appear to be a separate disease entity, which was also included in an adapted prognostic staging system [2, 3].

HPV-induced HNSCC goes along with an integration of the viral genome into the genome of the host; this leads to a change in immune response and/or in the expression of cancer-related genes [4, 5]. Thereby, two biologically distinct HPV-related HNSCC subtypes exist alongside with three HPV-negative ones [6]. Accordingly, several molecular differences exist between HPV-positive and HPV-negative tumors, including higher frequency of intratumoral B cells in HPV-positive [7] and higher frequency of dysfunctional CD8+ T cells in HPV-negative patients [8]. Furthermore, tumors with HPV-positive entity show a similar pattern of numerical but a difference in structural chromosomal aberrations compared to HPV-negative ones [9]. While HPV-negative HNSCC often show mutations in genes like TP53 and CDKN2A, which is primarily associated to tobacco, HPV-positive tumors represent an increased expression of the E6 and E7 viral oncoproteins, resulting in a degradation of p53 and functional inactivation of Rb [10]. These molecular and immunological differences lead to several clinical consequences. Thereby, HPV-positive tumors often show large metastases of cervical lymph nodes [11]. However, one important issue related to outcome and therapy of the tumor is the survival rate, which has been reported to be improved in HPV-positive compared to HPV negative entities [4].

Altogether, the understanding of HPV-positive HNSCC, related genetic mechanisms and risk prediction is of high clinical interest to improve the understanding of pathogenesis and to choose the appropriate therapy. Thereby, different studies aimed in the detection of potential hub genes in this context [4, 6, 7, 10, 12–15]. Improving the knowledge on and understanding of the potential role of HPV integration in HNSCC biology appears thereby fundamental with regard to the design of new therapeutic strategies and selection of patients for individual therapeutic measure [4]. Thus, especially the understanding of the immunologic as well as genomic landscape of HPV-related HNSCC is expected to be helpful to improve strategies to stratify patients for the respective therapy [10]. This approach is of relevance to focus on personalized medicine and patient-centered, individualized cancer therapy.

Accordingly, this current study aimed in the assessment of the viral- and carcinogen- driven HNSCC microenvironment based on single-cell sequencing analysis. Thereby, HPV-positive and HPV-negative entities should be analyzed, and infiltrating immune cells were examined in this respect. Thereby, the differentially expressed genes should be investigated regarding their relationship to survival and their location of expression.

## 2. Materials and Methods

### 2.1. Dataset Acquisition

The gene barcode matrix of scRNA-seq was obtained through the GSE139324 dataset [14, 16] in the Gene Expression Omnibus (GEO) database [17]. The data contained 131224 cells that were derived from 63 samples, of which 26 samples each were paired peripheral blood mononuclear cells (PBMC) and tumor infiltrating immune cells from HNSCC patients (18 HPV- and 8 HPV+), 6 were PBMC from healthy donors, and 5 were tissue resident immune cells from healthy donor tonsils. The Seurat package (version 3.0) [18] in R program (version 4.0.3) was used to perform data analysis including quality control, dimensional reduction, and visualization.

### 2.2. Single-Cell Data Preprocessing

Batch effects were mitigated by the Harmony package when data integration was performed. The SCTransform method [19] was adopted for data normalization. Following data normalization, all highly variable genes in single cells were identified based on high dispersion and checked for the relationship between expression magnitude and variance. The top 2000 genes by dispersion were carried forward into scaling and principal component analysis (PCA). Prior to PCA, unwanted sources of variation (e.g., the number of genes per cell, percentage of mitochondrial genes per cell, and percentage of ribosomal genes per cell) were controlled using an approach implemented in Seurat, resulting in scaled and centered corrected values. In detail, low-quality cells were excluded, based on two quality measures: (1) for each gene, it should be expressed in at least three cells, and the number of genes with expression should be more than 200 per cell, and (2) the percentage of mitochondrial genes should be less than 15, and the ribosome gene percentage of more than 20 was filtered.

Subsequently, a principal component analysis (PCA) was used with variable genes as the input and significant principal components (PCs) were identified based on the jackstraw function. Thirty PCs were selected as the input for uniform manifold approximation and projection (UMAP) and t-distributed stochastic neighbor embedding (tSNE), when statistically significant. Identified clusters of cells by a shared nearest neighbor (SNN) modularity optimization were analyzed based on the “matrix” clustering algorithm [20].

### 2.3. Major Canonical Immune Lineages and Cluster Markers Identification

Differentially expressed genes between clustering were identified using a Wilcoxon rank sum test, comparing natural log transformed and library size normalized expression values between the cluster of interest and all other clusters, or between two targeted clusters. Genes were required to be expressed in 10% of cells in each cluster with an average log-fold change greater than 0.25 to be considered differentially expressed, as implemented in Seurat [18]. The FindAllMarkers function [21] was used to list the markers of each cell cluster. The major cell types were then recognized based on the markers obtained from the CellMarker database [22] and SingleR database [23].

### 2.4. Functional Enrichment Analysis

A gene functional enrichment analysis was performed based on the marker genes in each cell cluster. These differential expressed genes were loaded into clusterProfiler [24] for the Gene Ontology (GO) enrichment analysis. Only gene sets with false discovery rate (FDR) *p* value less than 0.05 and nominal *p* values less than 0.05 were considered significantly enriched.

### 2.5. HPV Types Present in Head and Neck Cancer Filtering and Survival Analysis

To assess the clinical significance of expression of a given gene set, RSEM normalized log2 bulk RNA-seq expression data available from The Cancer Genome Altas (TCGA, URL: https://www.cancer.gov/about-nci/organization/ccg/research/structural-genomics/tcga) [25] through the Firehose were utilized. Moreover, clinical and outcome data, available through the published Pan-Cancer Clinical Data from TCGA, were included. From these resources, the analysis was filtered by patients that underwent testing for HPV status via p16 immunohistochemistry. Five-year overall survival outcomes were compared only in both HPV+ and HPV- subsets of HNSCC patients, dichotomized by median expression for all metabolic genes. Log-rank statistical *p* values were calculated for each Cox survival model. The derived log-rank *p* values for all tested genes were assessed for significance after correcting for false discovery rate (FDR) using the Benjamin-Hochberg method, and an FDR threshold of 0.1 was set for significance. Univariate analysis was performed through R (version 4.0.3) based on a Cox Proportional Hazard Model [26] using the survival package [27]. Stepwise bidirectional multivariate analysis was then carried out with clinical variables, including gene expression level and HPV type.

### 2.6. Cell–Cell Communication Analysis

The CellChat v1.1.3 software [28] was used to infer cell–cell communication based on ligand–receptor interaction with default parameters. For each ligand–receptor pair, only the secreted signaling interaction category was considered for downstream analysis. The cell–cell communication was filtered out if there are fewer than 15 cells in certain cell groups. The statistical significance of communication probability values was assessed by a permutation test. *p* < 0.05 was considered statistically significant.

## 3. Results

### 3.1. Immune Landscape of Patients with HNSCC at Single-Cell Resolution

**Figure 1:**
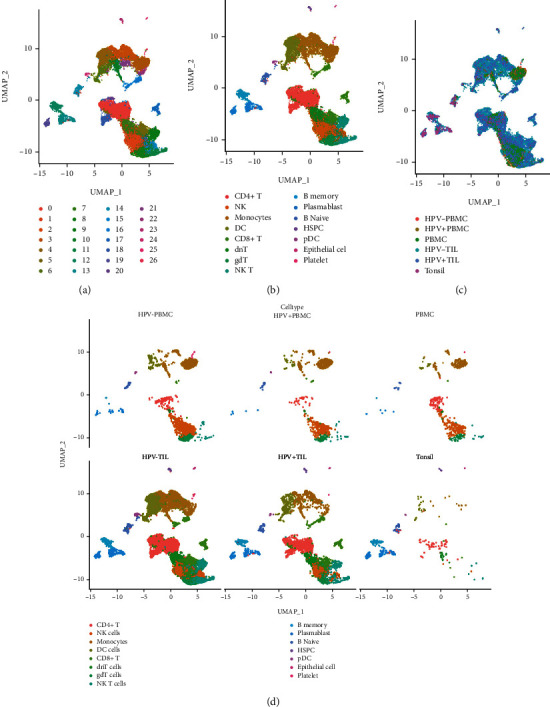
Single-cell RNA sequencing results from immune cells in HNSCC. (a) UMAP of 20,978 primary immune cells from peripheral and intra-tumoral CD45+ immune populations from HPV- and HPV+ HNSCC and healthy donors. (b) Cell cluster identification across the UMAP. (c) Distribution of cells by tissue type, peripheral blood from HPV-, tonsil from HPV-, peripheral blood from HPV+, tonsil from HPV+, peripheral blood from healthy donors, and tonsil from healthy donors. (d) UMAP demonstrating inferred immune cell types in HNSCC and healthy donors; clusters are colored by cell type across tissue types.

In order to define the immune microenvironment of HNSCC, single-cell RNA sequencing analysis was performed on the samples from different origin. In detail, the sample data were derived from paired blood and tumor-infiltrating leukocytes (TILs) obtained from 18 HPV- HNSCC patients, 8 HPV+ HNSCC patients, 5 tonsil tissues from independent patients without cancer, and peripheral blood mononuclear cells (PBMC) from 6 healthy donors. In total, 20,978 high-quality individual cells passed quality control measures. All high-quality cells were integrated into an un-batched and comparable dataset and subjected to principal components analysis (PCA) after correction for read depth and mitochondrial read counts. By graph-based uniform manifold approximation and projection (UMAP), 27 clusters across 20,978 cells primary immune cells were identified (Figure 1(a)).

Based on gene expression, cell lineages were assigned to each cluster using a two-method approach: (1) gene expression of canonical markers for platelet cells (PPBP), epithelial cells (SPRR3), pDC cells (LILRA4), HSPC cells (TPSAB1), B naïve cells (MS4A1), plasmablast (IGLC2), and natural killer (NK) cells (NKG7) and (2) correlations with gene signatures were derived from purified cell populations deposited by ENCODE. Based on these approaches, clusters as CD4+ T cells (cluster 0, 18, 20), NK cells (cluster 1,15), monocytes (cluster 2, 3, 10, 21, 22), DC cells (cluster 4, 6), CD8+ T cells (cluster 5, 16, 17), dnT cells (cluster 7), gdT cells (cluster 8), NK T cells (cluster 9, 11), B naïve cells (cluster 14), B memory cells (cluster 12), Plasmablast cells (cluster 13, 19), pDC cells (cluster 24), HSPC cells (cluster 23), epithelial cells (cluster 25), and platelet cells (cluster 26) were annotated (Figure 1(b)). Despite the integration of sequencing runs to reduce tissue-type divergence, each tissue type had enrichment for distinct clusters: Tumor TIL tissues were mainly enriched within Clusters 2, 4, 5, 6, 7, 9, 11, 16, and 17, while peripheral blood derived from matched tumor patients was enriched within Cluster 3, and normal tonsil tissues formed the majority of Cluster 12, 13, 14, and 19 (Figure 1(c)).

To identify the differences among cell clusters of these major cell types, the cells derived from different tissues were clustered separately. Across the tissues differently derived, TIL (including 3821 cells originated from HPV+ TIL HNSCC tumor, 12819 cells were HPV- TIL HNSCC, 467 cells derived from healthy tonsil tissues) and PBMC (including 2223 cells obtained from HPV- PBMC, 988 cells derived from HPV+ PBMC, and 660 cells originating from healthy donors), a number of clusters sharing similar gene expression were found. Distribution of cells showed a clear difference between TIL derived from HNSCC patients and PBMC (HNSCC-PBMC and healthy PBMC). It was noticed that immune cells, especially DC cells, monocytes, CD4 + T cells, CD8 + T cells, NK cells, and gdT cells, were highly enriched in tumor tissues (HPV-TIL and HPV + TIL). In addition, tumor-derived DC cells and monocytes were highly tumor tissue specific, while the cell abundance in PBMC from HNSCC patients was much higher (Figure 1(d)). This indicates that the immune microenvironment varies between different types of tissues.

### 3.2. Similar and Distinct Cell Components and Distribution in HNSCC

**Figure 2:**
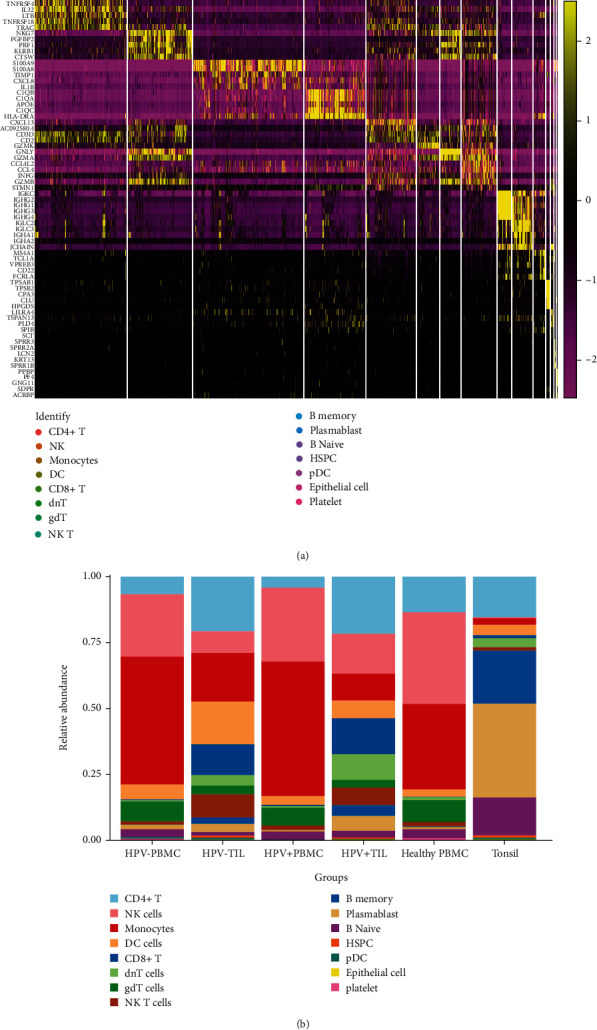
Analysis of the cell population and distribution differences in different types of tissue. (a) Heatmap plot showing selection of cell type–specific markers across major clusters. The color of the sample represents the relative gene expression level. (b) Bar plot of cell composition in different groups across cell types and sample sources.

To better understand the populations of cell clusters, the top five differentially expressed genes across clusters were investigated. The top five markers of the main cell clusters were visualized as a heatmap (Figure 2(a)). Clearly, most of cell cluster had its specific expressed genes, while some of clusters showed a similar gene expression tendency, including CD8 + T cells, dnT cells, gdT cells, and NK T cells, that might be owing to the similar function of these cell clusters.

To be more comprehensive and intuitive in observing changes in cell composition across different groupings, cell components were visualized in the form of bar plot between cell types and sample sources (Figure 2(b)). The abundance of NK cells, monocytes, and CD4 + T cells were the first top three cell types in PBMC derived from healthy donors. However, there was a clearly decreasing population of CD4 + T cells compared with HNSCC-PBMC samples, and the abundance of dnT cells increased obviously, ranking as the new third one in HNSCC-PBMCs (including HPV-PBMC and HPV + PBMC). In tonsil tissue, the top three cell types were plasmablasts, CD8 + T cells and B naïve cells, while in TIL derived from HNSCC patients, the population of CD4 + T cells, DC cells, and monocytes were the top three. Even though in HNSCC patients, the cell populations between PBMC and TIL also showed a great difference. A greater decrease of monocytes and NK cells within tumors (HPV-TIL and HPV + TIL) compared to peripheral blood (HPV-PBMC and HPV + PBMC) could be observed. Conversely, an increase of CD4+ T cells, CD8 + T cells, DC cells, and NK T cells in tumors (HPV-TIL and HPV + TIL) in comparison to peripheral blood (HPV-PBMC and HPV + PBMC) was noticeable. Notably, in HNSCC patients, the cell abundant of monocytes and DC cells derived from HPV-TIL was higher than that in HPV + TIL. However, NK cells and CD8 + T cells were significantly enriched in HPV + TIL, and the platelet cells were almost only detected in HPV-PBMC and HPV-TIL (Figure 2(b)).

### 3.3. CellChat Identifies Communication Patterns and Predicts Functions in HNSCC

Single-cell transcriptomic analysis not only reveals cell intrinsic information but can also probe putative cell extrinsic interactions through interrogation of ligand and receptor expression [29]. When cells do not properly interact or improperly decode molecular messages, this indicates status of disease. Intercellular connections are an important pathway for cell–cell crosstalk and ligand–receptor pairs, which can be used to infer intercellular communication from the coordinated expression of their cognate genes. To investigate possible cell–cell interactions, analysis with CellChat R package was performed [28].

**Figure 3:**
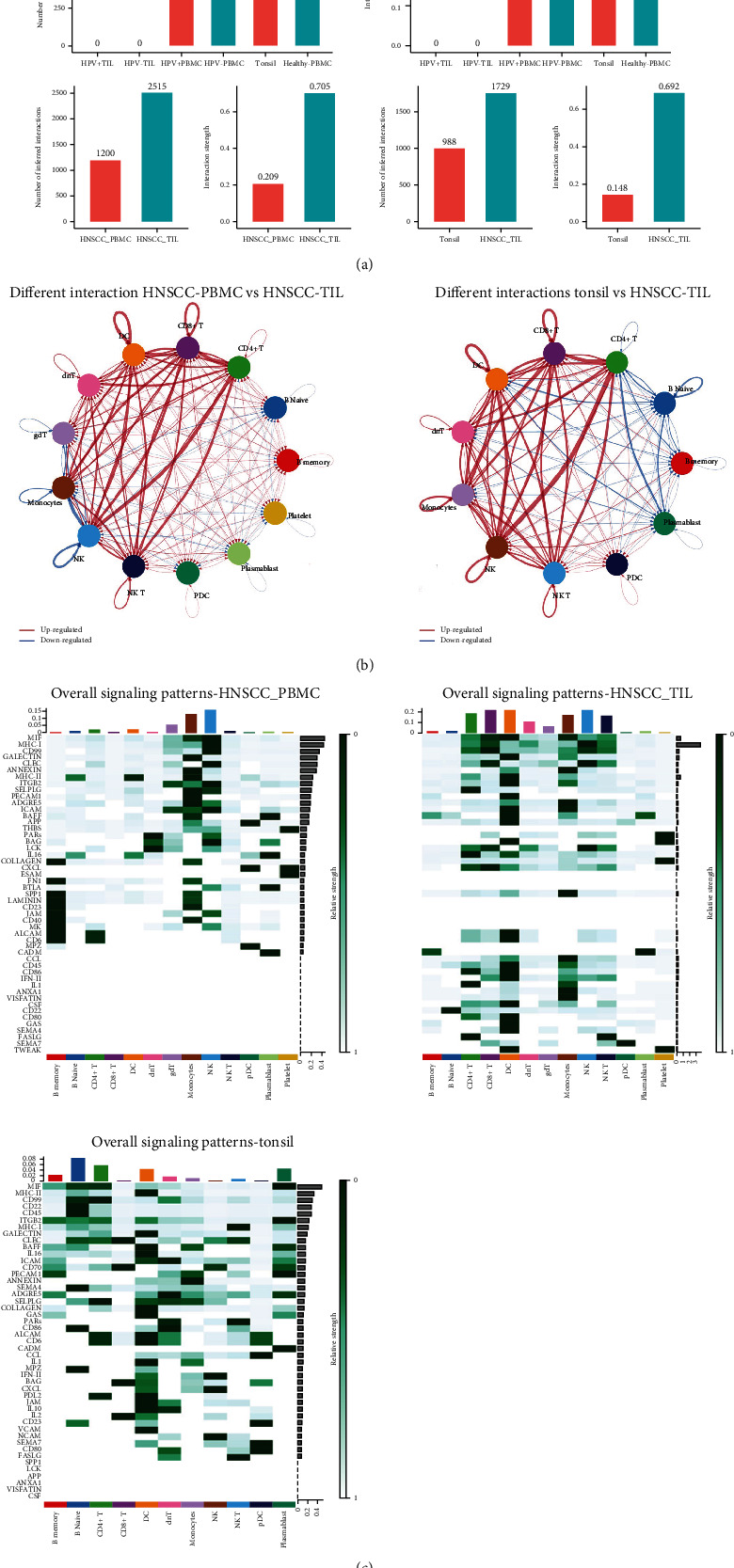
Cell-Cell interaction comparison in different types. (a) Overview on the different interactions in HNSCC-TIL, HNSCC-PBMC, normal tonsil, and healthy PBMC. Left side is the interaction number, and right side represents interaction strength. (b) Visually integrated cellular communication network. Circle chart showing the interaction between any two cell groups. (c) Overall signal patterns in different cells derived from HNSCC-PBMC, HNSCC-TIL, and tonsil in the heatmap; the vertical axis is the cell that sends or receives the signal, and the horizontal axis is the pathway that receives or sends the signal. The color of the heat map represents the strength of the signal. The pillars on the upper and right sides are the accumulation of the strength of the vertical axis and the horizontal axis.

First, CellChat analysis on the integrated dataset was applied to compare the cell-cell interactions derived from different types, which include number of interactions and interaction strength (Figure 3(a)). When samples were separated into six groups (HPV-TIL, HPV + TIL, HPV-PBMC, HPV + PBMC, tonsil, and healthy PBMC), no interaction in HPV-TIL or HPV + TIL were detected. While the interaction strength in HPV-PBMC was the highest, followed by healthy PBMC, and tonsil, the interaction strength in HPV + PBMC was the least strong. Next, the cell–cell interactions only on PBMC and TIL samples, which derived from HNSCC, were compared. The results show a clearly high number of interactions in HNSCC-TIL. Similarly, the number of inferred interactions was much higher in HNSCC-TIL compared to normal tonsil. This indicates that the signaling crosstalk in cells varies under different conditions, which is critical for informing diverse cellular decisions.

In addition to explore detailed communications for individual pathways, an important question is how multiple cell groups and signaling pathways coordinate to function. Therefore, the differential status of interactions in different groups was investigated: (1) HNSCC-PBMC and HNSCC-TIL and (2) normal tonsil and HNSCC-TIL. Compared with HNSCC-PBMC or normal tonsil, most of the interactions among CD4 + T cells, DC cells, dnT cells, gdT cells, monocytes, NK cells, and NK T cells were upregulated, and the interactions among these immune cells were stronger. This indicates that the immune response in HNSCC-TIL is much more stronger. Then, the overall (including outgoing and incoming) signaling patterns in HNSCC-PBMC, HNSCC-TIL, and normal tonsil were compared (Figure 3(c)).

The output of this analysis is a set of the so-called communication patterns that connect cell groups with signaling pathways either in the context of outgoing signaling (i.e., treating cells as senders) or incoming signaling (i.e., treating cells as receivers). The results clearly show which signals contribute most to the outgoing or incoming signals of certain cell groups. For example, the MIF signal strength was most strong in B naïve cells and platelets derived from normal tonsil, but the signal pattern becomes stronger in monocytes and DC cells derived from HNSCC-TIL, and the signal strength almost disappeared in most of cells except for monocytes and NK cells derived from HNSCC-PBMC.

From overall signal patterns, including outgoing and incoming signals, monocytes and NK cells were the two dominant cell types in cell–cell communication, and the signal strength of MIF and MHC-I pathways ranked as the first top two. Compared with HNSCC-TIL, except for monocytes and NK cells, the signal strength in certain cell types becomes stronger, including CD4 + T, CD8 + T, DC cells, dnT cells, gdT cells, and NK T cells. However, the signal strength in most of pathways sharply decreased, and only MNC-I pathway had the highest accumulation strength across all the immune cells. This indicates that the MHC-I pathway plays vital role in immune response. Furthermore, in normal tonsil, most of the relative strength of cell clusters was slightly weaker, and the top two cell types containing strongest signal strength were B naïve and CD4 + T cells. The pathways that have strong accumulation signal strength were MIF and MHC-II. Overall, the MHC-I, MHC-II, and MIF signal pathways were the most important three in cell–cell communication, and the different signal strengths might reflect the disrupted process during the immune response.

### 3.4. Comparison of Cell-Cell Communication Patterns in Key Signal Pathways in Different Tissue Groups

**Figure 4:**
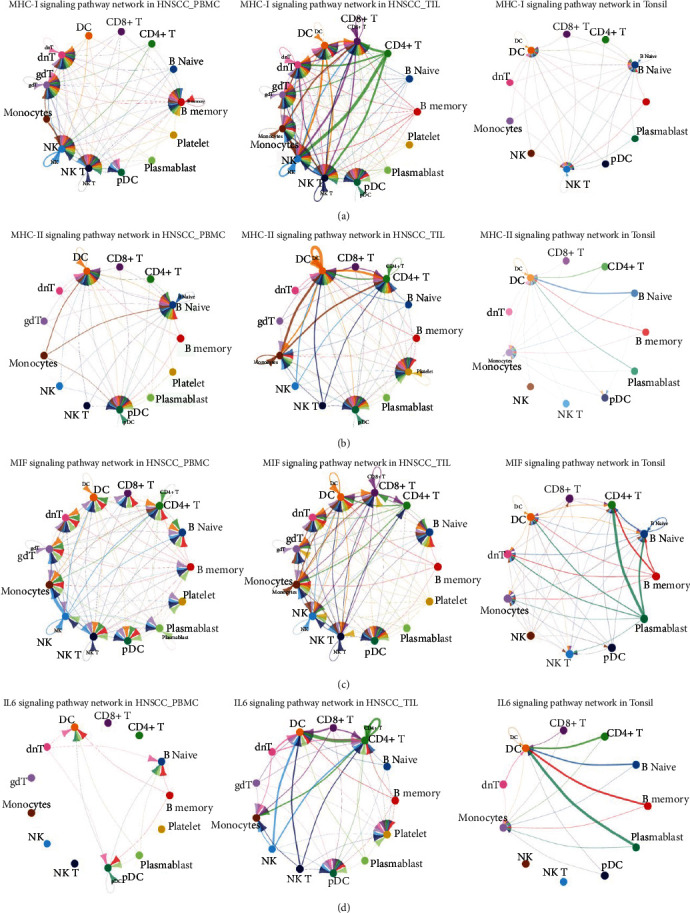
Specific signaling events In the signal pathway network. (a)MHC-I signaling pathway network in HNSCC PBMC, TIL, and tonsil. (b) MHC-II signaling pathway network in HNSC PBMC, TIL, and tonsil. (c) MIF signaling pathway network in HNSC PBMC, TIL, and tonsil. (d) IL6 signaling pathway network in HNSCC PBMC, TIL, and tonsil. The edge color is consistent with the sender's source, and the edge weight is proportional to the interaction strength. A thicker edge line indicates a stronger signal. In the circle chart, the size of the circle is proportional to the number of cells in each cell group.

Next, focus was set on the three key signal pathways, including MHC-I, MHC-II, and MIF, to compare their cell–cell interaction differences among HNSCC-PBMC, HNSCC-TIL, and normal tonsil. There were more signal receivers in HNSCC-TIL than in HNSCC-PBMC or normal tonsil. In HNSCC-TIL and normal tonsil, B memory cells were signal senders, while in HNSCC-PBMC, B memory cells were the signal receiver. Conversely, monocytes were the signal receiver in HNSCC-PBMC, but they became a signal receiver in HNSCC-TIL. The number and strength of cell–cell interactions in normal tonsil was lower than that in TIL or PBMC derived from HNSCC (Figure 4(a)). The cell communications in MHC-II signal pathways became less than in MHC-I. In HNSCC-PBMC, B naïve cells and monocytes were the signal receivers or senders; however, the roles of these cells were conversed in HNSCC-TIL, where B naïve cells function as signal senders and the number of interactions was decreased. The monocytes became signal receivers, and the signal strength associated with monocytes was much stronger. In addition, the role of CD4 + T cells switched from sender in normal tonsil to receiver in HNSCC-TIL (Figure 4(b)). For the MIF signaling pathway, B memory cells, platelets, and plasmablasts were the only three signal senders in HNSCC-TIL. This was conversely with HNSCC-PBMC, while CD8 + T cells became signal senders in normal tonsil from receivers in HNSCC-TIL (Figure 4(c)).

**Figure 5:**
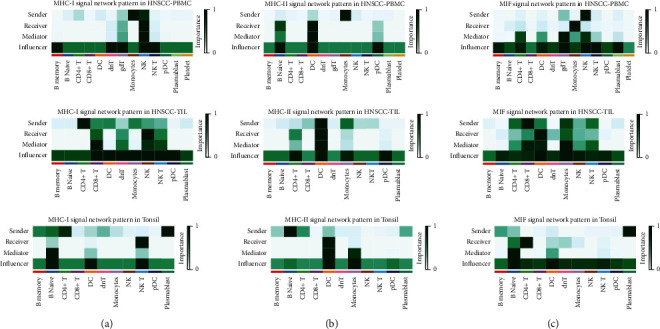
Identification of the main contribution signals of the cell group in the network. (a) MHC-I signal network pattern in HNSCC PBMC, TIL, and tonsil. (b) MHC-II signal network pattern in HNSCC PBMC, TIL, and tonsil. (c) MIF signal network pattern in HNSCC PBMC, TIL, and tonsil. The color in the heatmaps indicates the relative strength of the signal among cells. The horizontal axis is the cell type, and the vertical axis is the main contribution signals, including sender, receiver, mediator, and influencer. Bars to the right correspond to signal strength.

In the following, the main senders, receivers, mediators, and influencers of cell-to-cell communication in the three signal pathways were identified. In a weighted directional network with weights as the communication probability, the out-degree was calculated as the sum of the communication probabilities of the outgoing signals from the cell group and calculated as the sum of the incoming signal to the unit group's communication probabilities, which can be used to identify the main unit element transmitter and receiver of the signal network (Figure 5). Even for the same pathway, the signal network patterns were different in distinct type conditions.

In MHC-I signal network, monocytes and CD4 + T cells were the main signal senders in HNSCC-PBMC and HNSCC-TIL separately; the main receivers in PBMC or TIL derived from HNSCC were NK cells. However, in normal tonsil, the signal senders were plasmablast cells, and receivers were NK T cells. Similarly with MHC-I signal network, the signal senders in MHC-II signal network of HNSCC-PBMC were monocytes, and main receivers were DC cells. While in HNSCC-TIL, the signal senders and receivers were mainly DC cells, and the senders and receivers in tonsil changed as B naïve and DC cells. In MIF signal network, the main senders were NK cells, CD8 + T cells, and plasmablasts in HNSC-PBMC, HNSCC-TIL, and normal tonsil separately. Conversely, the signal receivers were monocytes, DC cells, and CD4 + T cells. Overall, in different types of conditions, the main contribution signals of the cell group were different. Changing the cell–cell interactions might play a vital role in regulating the immune response.

### 3.5. HPV+ HNSCC Patients Have better Overall Survival Compared with HPV- HNSCC Patients

**Figure 6:**
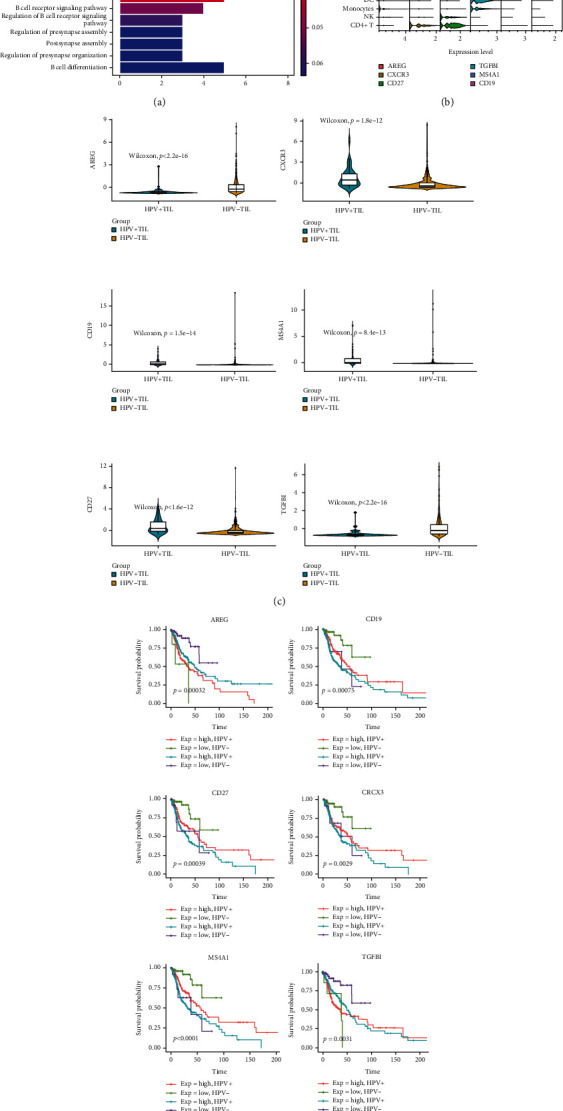
Marker genes associate with prognostic signatures. (a) Biological process enrichment analysis on differentially expressed genes between HPV-TIL and HPV + TIL groups. (b) Violin plot of marker genes in cell clusters in single-cell resolution. (c) Differentially expressed genes comparison between HPV-TIL and HPV + TIL groups based on TCGA data. (d) Overall survival analysis on six marker genes based on HPV types and gene expression levels.

RNA-seq data were used from 522 HNSCC primary tumor samples from the TCGA to comprehensively compare the expression of genes between HPV+, HPV-, and normal-adjacent control tissues. The number of HPV+ and HPV- type of HNSCC tumor samples were 73 and 449, respectively. In total, 1,137 differentially expressed genes between HPV+ and HPV- HNSCC patients were investigated, including 646 upregulated and 491 downregulated genes in HPV-TIL compared with HPV + TIL samples. There were 96 genes, which were differentially expressed between the two types of tumors and associated with overall survival signatures. Biological process enrichment analysis showed that these genes were highly enriched in B cell associated processes, including B cell activation, B cell proliferation, B cell receptor signaling pathway, and B cell differentiation, indicating that the activity of B cells might be essential for the prognostic prediction (Figure 6(a)). Six among the 96 genes expressed differentially between HPV-TIL and HPV + TIL samples were marker genes in specific cell clusters, including AREG, TGFBI, CD27, CXCR3, MS4A1, and CD19. (Figures 6(b)–6(c)). Furthermore, overall survival analysis on those six genes showed that the gene expression level and HPV types were highly associated with survival prognostic signature (Figure 6(d)).

Meanwhile, through literature searching and database mining, all of these six genes were found to be marker genes related to HNSCC. The current analysis results further confirmed the differential expression of these 6 genes in different HPV types of HNSCC, and also these 6 genes were significantly related to prognostic survival. Among these six genes, AREG and TGFBI were upregulated in HPV-related HNSCC, and the other four genes were downregulated in HPV-related HNSCC (Table 1).

By analyzing the cell location of these 6 genes, it can be found that the relationship between the 6 genes and HPV type may depend on the specific expression of these genes in B cells and T cells. The corresponding results showed the following: Both AREG and TGFBI were pDC marker genes; CXCR3 and CD27 were significantly expressed in T cell-related cells, while MS4A1 and CD19 were mainly expressed in B naïve cells. This further indicates that B cells and T cells within the tumor microenvironment were highly correlated with overall survival across human tumor types.

## 4. Discussion

The current study included a variety of complex analyses to reveal the viral- and carcinogen-driven HNSCC microenvironment based on single-cell analysis. As main findings, 1,137 differentially expressed genes between HPV+ and HPV- HNSCC were revealed, whereby 96 differentially expressed genes were associated with survival. Overall, the cell composition as well as their role as sender or receiver differed between sample types. MHC-I, MHC-II, and MIF signaling pathways were found to be most relevant. Six genes, i.e., AREG and TGFBI (upregulation), CD27, CXCR3, MS4A1, and CD19 (downregulation), were found, whose expression and HPV types were highly associated with worse overall survival. In the following, the discussion will focus on several main outcomes of the current analysis.

In the current study, differences in cell composition in different types of samples were observed. In HNSCC patients, the cell populations between PBMC and TIL were quite different. Differences between PBMC and TIL are not surprising; a recent study comparing the immune checkpoint expression on T-cells of peripheral and tumor-infiltrating lymphocytes also found clear differences [30]. In this previous study, immune checkpoint expression was increased in TIL in CD8+ as well as CD4+ T-cells [30]. Increased CD4+ and CD8+ was found in TIL compared to PBSC in the current study, indicating that these immune cells would be more specialized and tumor-reactive tissues, caused by their exposure to tumor microenvironment. Thereby, a shift appears to occur, as a decrease of monocytes and NK cells is observed within tumors (HPV-TIL and HPV + TIL). Moreover, cell abundant of monocytes and DC cells derived from HPV-TIL was higher than that in HPV + TIL, while NK cells and CD8 + T cells were significantly enriched in HPV + TIL. The HPV-related immune reaction, especially the activation of IFN*γ* or IL-17 producing CD8+ T cells [31], might be an explanation for these findings. Thereby, a recent clinical study also found differences in immune cell infiltration between HPV positive and negative samples, although these findings differed from the current results [32].

Furthermore, when the samples in the current study were separated into six groups (HPV-TIL, HPV + TIL, HPV-PBMC, HPV + PBMC, tonsil, and healthy PBMC), no interaction in HPV-TIL or HPV + TIL was detected. A HPV infection leads to complex immunological processes, involving different mechanisms and strategies to thwart or infiltrate immune response of the host [4, 33, 34]. Thereby, HPV drives cancer immune escape [33]. During these immunological processes, HPV may restrict the expression of receptor or ligand genes so that only ligands or receptors can be detected in HPV+/-; therefore, no intercellular signaling pathways are detected in the current study. Until now, this remains a hypothesis because the underlying mechanisms remain speculative and are not fully understood, yet.

Three key signaling pathways were further examined in the current study, including MHC-I, MHC-II, and MIF. Thereby, some cells were signal receiver in one kind of sample; however, these cells became signal sender in another type of sample. This argues for a sample specific immune reaction and a difference between TIL and PBMC, which has already been stated above. MHC-I, i.e., major histocompatibility complex class I is a protein, which is expressed on cell surfaces, mediating CD8+ T-cell activation and regulating the activity of NK cells [35]. Accordingly, MHC-I is a mediator of immunosurveillance against cancer playing a role in carcinogenesis [35, 36]. Therefore, MHC-I has already been confirmed to be of relevance in HNSCC pathogenesis [37, 38], as well as HPV-related carcinogenesis [37, 39]. In this current analysis regarding MHC-I, more signal receivers in HNSCC-TIL were found than in HNSCC-PBMC or normal tonsil. This again argues for the hypothesis that the tissue immune cells would be more specialized and tumor-reactive. The current finding shows that B memory cells were signal senders in HNSCC-TIL and normal tonsil, while B memory cells were the signal receiver in HNSCC-PBMC. Such finding would indicate an inverse reaction between tissue and blood. MHC-II, i.e., major histocompatibility complex class II, has been reported to restrict CD4+ T-cell response and thus also to be relevant in tumor immunity [40]. It has been shown that there are non-overlapping functions in antitumor response between MHC-I and MHC-II [40]. Therefore, it is not surprising that a difference in cell communication between these two pathways was found in the current study, where the cell communications in MHC-II signal pathways were less than in MHC-I. Similar as for MHC-I, however, an inverse role of B cells and monocytes between tissue and PBMC was found for MHC-II. Interestingly, CD4 + T cells move from sender in normal tonsil into receiver in HNSCC-TIL that argues for an alteration in cancer-related immune response in this context. Thereby, the importance of CD4+ T-cells in senescence and cancer surveillance in context of cancer immunosurveillance [41] is supported in the current study samples. Furthermore, the macrophage migration inhibitory factor (MIF) is known to enable tumor macrophages to be polarized to an immunosuppressive and thus pro-tumorigenic phenotype [42]. In this respect, HNSCC cells were found to lead to MIF-dependent macrophage polarization [43]. Similarly as for MHC-I and MHC-II, opposite roles of B-cells were found between tissue and blood. Moreover, the role of CD8 + T cells switched from signal senders in normal tonsil to receivers in HNSCC-TIL. It has been shown that blocking MIF-related signaling potentiates CD8+ T-cell infiltration [44]. Thus, this switch also indicates a change in the immunoinflammatory environment. This approach is also supported by the findings shown in figure 5, where respective signal networks of the three pathways are displayed. CD4 + T-cells are the main signal senders in HNSCC-PBMC and HNSCC-TIL in MIH-I associated network, and CD8 + T-cells are the main signal senders in HNSC-PBMC, HNSCC-TIL, and normal tonsil in MIF associated network. This supports the important roles of CD4+ and CD8+ T-cells in the tumor microenvironment of HNSCC, as indicated in recent literature [14, 45]. Thereby, regulation of T-cell proliferation appears crucial for carcinogenesis, although the underlying mechanisms are not fully understood, yet. The current study's findings provide therefore detailed information on the related pathways in different sample types, indicating a complex interrelationship between immune cells acting as signal senders or receivers in the respective habitat.

This comprehensive examination based on single-cell sequencing analysis revealed patients with HPV-positive tumors to have an improved survival, whereby the related genes were primarily connected to B-cell associated pathways. Thereby, the upregulation of AREG and TGFBI, which were pDC marker genes, was associated with worse survival. Furthermore, the higher expression of CD27 and CXCR3, which were significantly expressed in T cell-related cells, as well as of MS4A1 and CD19, which were mainly expressed in B naïve cells, was associated with improved survival.

Overall, the presence of the potential impact of an HPV infection on the outcome of HNSCC has been repeatedly discussed in literature; it has thereby been shown that the survival is better in HPV-positive patients with a respective tumor [4, 46–48]. This is in line with the current study's findings, which are therefore not surprising in this respect. To gain a deeper understanding of HPV-related outcome of HNSCC, the six identified genes will be discussed in the following.

AREG, which is the abbreviation for amphiregulin is a ligand of the epidermal growth factor receptor; by binding on that receptor, AREG can activate different signaling cascades, which are important in governing cell survival, proliferation, and motility, explaining its potential relevance in neoplastic diseases [49]. Recently, AREG has been reported to be related to the outcome of different therapeutic strategies of HNSCC [50–53]. It has been demonstrated in vitro that AREG is targeted by miR-34a, whereby the increased expression of AREG was related to cell invasion and metastatic potential of HNSCC [54]. Hsieh et al. reported that AREG would be related to the resistance of HNSCC against vincristine and thus to the failure of a respective therapy with this drug [50]. The HPV status appears to be related to the therapeutic outcome with regard to AREG; it was shown that HPV-positive HNSCC showed increased susceptibility to sorafenib and sunitinib, leading to a suppression of AREG expression [53]. Additionally, the HPV status influenced the treatment response to tyrosine kinase inhibitors with regard to AREG expression, underlining the necessity to consider HPV infection for therapy of HNSCC [52]. Accordingly, the relationship between AREG expression and survival is plausible due to its influence on metastatic potential and therapy response, making AREG a potential marker for survival and therapeutic options in HPV-positive individuals. The second pDC marker gene, whose overexpression was related to worse survival, was TGFBI. The transforming growth factor-beta-induced protein (TGFBI) is a protein of the extracellular matrix; the role in tumorigenesis is undeniable, while the data are quite convincing, because TGFBI has been shown to be both, a tumor suppressor as well as promoter, depending on the tumor microenvironment [55]. With regard to oral squamous cell carcinoma, TGFBI was reported to potentially alter cell response to bacterial stress, leading to an imbalance of the inflammatory environment, promoting cancer development [56]. Thereby, TGFBI was supposed to be a hub gene for HNSCC and to be associated with metastasis and worse prognosis [56]. Similar as AREG, TGFBI is also related to epidermal growth factor receptor (EGFR) signaling and is potentially related to therapy response, e.g., with respect to the resistance to cetuximab treatment [57]. Thereby, the epithelial-mesenchymal transition, which is related to the transforming growth factor B, is seen as a crucial issue in epithelial cancer development and metastasis [58]. This appears also of relevance in squamous cell carcinoma of the tongue [59]. Thus, the relationship between TGFBI overexpression and worse outcome appears plausible and could indicate that AREG and TGFBI and its relationship to EGFR might be a reasonable approach in therapy of HPV-associated HNSCC.

Cluster of differentiation 27 (CD27) plays an important role in T-cell activation; due to binding with its natural ligand CD70, T-cell proliferation and differentiation to effector and memory T-cells is enhanced, making it a potential target in cancer therapy [60]. Both CD27 and CD70 have been reported to be of relevance in HNSCC pathogenesis [61–65]. CD27 negative T-cells were found to contain precursors of interferon gamma producing T-cells and to be sensitive to apoptosis in venous blood samples of HNSCC patients [64]. A recent longitudinal study of immune checkpoint molecule expression of patients during HNSCC therapy found that CD27 agonistic antibodies could be a promising therapeutic strategy [62]. This is supported by the result of the current study that increased CD27 expression would be related to improved prognosis. The second gene that was significantly expressed in T cell-related cells was CXCR3, which is a G-protein coupled receptor with the ability to affect immune responses, vascular genesis, wound repair, and carcinogenesis, whereby a negative relationship to prognosis has been discussed [66]. CXCR3 is involved in the direction of CD4+ and CD8+ T-cells in context of cancer and autoimmunity [67]. In HNSCC, CXCR3 was reported to mediate a cross-talk between lymphatic endothelial cells and HNSCC, also showing a positive correlation between CXCR3 expression and lymphovascular tumor invasion [68]. Similar as TGFBI, CXCR3 is also involved in the induction of epithelial-mesenchymal transition and thus in promoting metastasis and invasion of tongue squamous cell carcinoma [69]. In cervical squamous cell carcinoma, the CXCL10-CXCR3 axis can be induced by a HPV infection, helping HPV to escape the immune response, predisposing carcinogenesis [70]. Altogether, the relationship between CXCR3 and potential lymphovascular invasion as well as metastasis might explain its relation to survival as revealed in the current analysis.

Additionally, two genes mainly expressed in B naïve cells were confirmed to be related to survival in the current study. The first one is membrane-spanning 4-domain family, subfamily A 1, i.e., MS4A1 and encoding CD20, respectively [71]. MS4A1 is a B-lymphocyte antigen, which is a one of the most commonly used biomarkers for tumor-infiltrating B-lymphocytes [72]. Its prognostic value for different cancers, including HNSCC is unclear, because literature is inconclusive regarding this issue [73]. In nasopharyngeal carcinoma, a higher expression of MS4A1 was associated to a longer progression free survival [74]. Similarly, MS4A1 was positively related to prognosis and therapy response in ovarian cancer [75]. Therefore, its potential to be a prognostic and therapeutically relevant biomarker appears given. With specific regard to the current study, the infiltration of MS4A1 positive B-cells was higher in HPV-positive patients with HNSCC that has been related to improved prognosis [63]. This is completely in line with the current study's findings. Moreover, the second B-cell related gene, i.e., CD19, has been found in the same context; CD19-positive plasma cells were more frequently infiltrated in HPV-positive HNSCC and related to improved prognosis, too [63]. CD19 is a co-receptor for B-cell antigen receptor signal transduction and a key player in regulation of cell proliferation as an important B-cell surface molecule [76]. CD19-positive B-cells were reported to be related to better response of PD-1 blockade in HPV-associated HNSCC and thus with improved survival [77]. Another study revealed CD19 to be one hub gene in HPV-related HNSCC, predisposing for a better therapy response and clinical outcome [12]. In addition, the presence of respective B-cells in tumor draining lymph nodes was related to better prognosis in HNSCC [78]. While for all of the six genes related with survival in HPV-positive HNSCC in the current study a plausible relevance can be striven, the two genes related to B-cell infiltration, i.e., MS4A1 and CD19, might have the most strongly relation to prognosis against the background of the currently available literature.

### 4.1. Strengths and Limitations

This current study aimed in the assessment of the viral- and carcinogen- driven HNSCC microenvironment based on single-cell sequencing analysis. Thereby, data of 20,978 high-quality individual cells underwent a comprehensive bioinformatics analysis, which revealed survival-associated genes in HPV-related HNSCC and their respective loci of expression. On that basis, an interpretation of these genes and related pathways regarding their potential as biomarkers and/or therapeutic targets was possible. Beside of these strengths, the main limitation is the absence of a clinical validation of the current study's results; although the analysis was very robust, a clinical validation remains necessary as a subsequent step. Each bioinformatics study is limited by that fact, and this must be considered in the interpretation of the hypotheses from the current study. Thereby, the applied single-cell analysis is limited with regard to the individuality of the different clinical patient cases, where the data originated. Therefore, it remains speculative in what extent the results reflect clinical reality or whether the results are primarily a statistical model. Accordingly, the findings serve a basis for future examinations in the field. In addition, all of the findings of that analysis are on transcriptomic level that needs to be recognized for the clinical translation of the findings.

## 5. Conclusions

This study based on single-cell sequencing revealed different regulation and expression of immune cells in tissue and blood samples of HNSCC. MHC-I, MHC-II, and MIF-related pathways, especially considering T-cell regulation by CD4+ and CD8+, appear particularly relevant in HNSCC, where different cells can act as senders and receivers of signals in different sample types. Six genes were revealed to be related to survival in HPV-related HNSCC. AREG and TGFBI (upregulation) as well as CD27, CXCR3, MS4A1, and CD19 (downregulation) were associated with worse overall survival in HPV-positive HNSCC. Especially B-cell-related pathways were revealed as particularly relevant in HPV-related HNSCC. These findings can provide a basis for the development of biomarkers and therapeutic strategies, needing further clinical validation.

## Figures and Tables

**Table 1:** The differential expression status of six marker genes related to HNSCC.

Gene symbol	Entreze ID	Log2FC	Adj. *P* value	HPV- vs. HPV+
AREG	374	2.816	7.72e-13	Up
CXCR3	2833	− 1.550	2.19e-13	Down
CD27	939	− 1.526	3.42e-16	Down
TGFBI	7045	1.710	5.00e-16	Up
MS4A1	931	− 2.83	1.20e-20	Down
CD19	930	− 2.543	2.6e-20	Down

## Data Availability

The data analyzed during the current study are available in the TCGA database with the accession numbers TCGA-HNSCC, and GEO repository (GSE139324 dataset).
